# 12-(2-Hy­droxy-6-oxo­cyclo­hex-1-en­yl)-9,10-di­hydro-8*H*-benzo[*a*]xanthen-11(12*H*)-one

**DOI:** 10.1107/S1600536813025324

**Published:** 2013-09-18

**Authors:** Mehmet Akkurt, Shaaban K. Mohamed, Alan R. Kennedy, Antar A. Abdelhamid, Gary J. Miller, Mustafa R. Albayati

**Affiliations:** a Department of Physics, Faculty of Sciences, Erciyes University, 38039 Kayseri, Turkey; b Chemistry and Environmental Division, Manchester Metropolitan University, Manchester, M1 5GD, England, Chemistry Department, Faculty of Science, Minia University, 61519 El-Minia, Egypt; c Department of Pure & Applied Chemistry, University of Strathclyde, 295 Cathedral Street, Glasgow G1 1XL, Scotland; d Chemistry and Environmental Division, Manchester Metropolitan University, Manchester, M1 5GD, UK; e Analytical Sciences, Manchester Metropolitan University, Manchester, M1 5GD, England; f Department of Chemistry, College of Science, Kirkuk University, Kirkuk, Iraq

## Abstract

In the xanthenone system of the title compound, C_23_H_20_O_4_, the pyran ring has a maximum deviation of 0.111 (1) Å from planarity and the outer cyclo­hexene ring exhibits a puckered conformation. The three methyl­ene C atoms of the cyclo­hexene ring bonded to the pyran unit are disordered over two sets of sites [occupancies = 0.570 (3) and 0.430 (3)]. In the crystal, mol­ecules are linked by C—H⋯O and O—H⋯O hydrogen bonds, forming a two-dimensional network parallel to (110). A C—H⋯π inter­action occurs between these networks.

## Related literature

For related xanthenone structures, see: Li *et al.* (2004 ▶); Abdelhamid *et al.* (2011 ▶); Mohamed *et al.* (2011 ▶, 2012 ▶). Reddy *et al.* (2009 ▶); Çelik *et al.* (2009 ▶). For the industrial and pharmaceutical significance of xanthenes, see: Zare *et al.* (2012 ▶); Menchen *et al.* (2003*a* ▶,*b* ▶); Sarma & Baruah, (2005 ▶). For ring conformations, see: Cremer & Pople (1975 ▶) and for standard bond lengths, see: Allen *et al.* (1987 ▶).
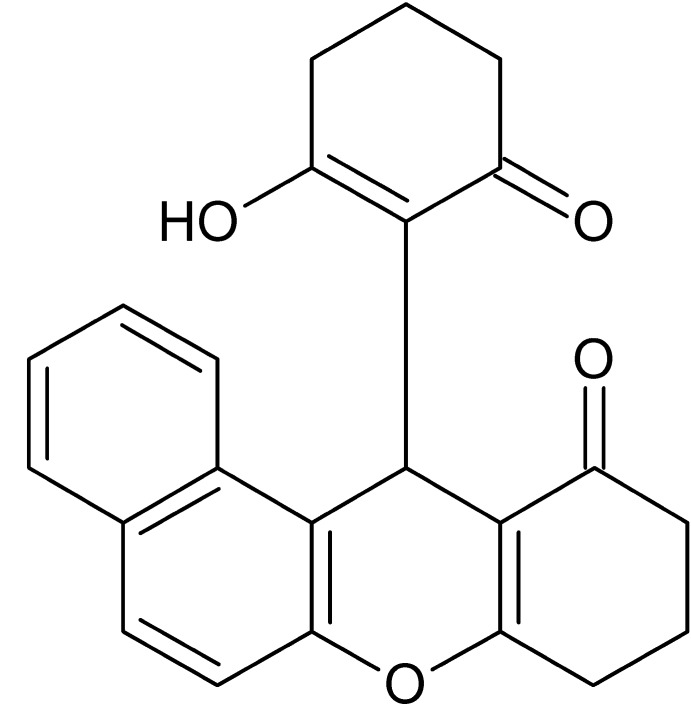


## Experimental

### 

#### Crystal data

C_23_H_20_O_4_
*M* *_r_* = 360.41
Orthorhombic, 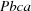

*a* = 14.2855 (15) Å
*b* = 13.7461 (12) Å
*c* = 18.400 (2) Å
*V* = 3613.2 (6) Å^3^
*Z* = 8
Mo *K*α radiation
μ = 0.09 mm^−1^
*T* = 123 K
0.20 × 0.18 × 0.16 mm

#### Data collection

Oxford Diffraction Xcalibur, Eos diffractometer
Absorption correction: multi-scan (*CrysAlis PRO*; Oxford Diffraction, 2010 ▶) *T*_min_ = 0.994, *T*_max_ = 1.000
17944 measured reflections
4541 independent reflections
3366 reflections with *I* > 2σ(*I*)
*R*_int_ = 0.042

#### Refinement

*R*[*F* ^2^ > 2σ(*F* ^2^)] = 0.051
*wR*(*F* ^2^) = 0.114
*S* = 1.04
4541 reflections
258 parameters
8 restraints
H atoms treated by a mixture of independent and constrained refinement
Δρ_max_ = 0.40 e Å^−3^
Δρ_min_ = −0.25 e Å^−3^

### 

Data collection: *CrysAlis PRO* (Oxford Diffraction, 2010 ▶); cell refinement: *CrysAlis PRO*; data reduction: *CrysAlis PRO*; program(s) used to solve structure: *SHELXS97* (Sheldrick, 2008 ▶); program(s) used to refine structure: *SHELXL97* (Sheldrick, 2008 ▶); molecular graphics: *ORTEP-3 for Windows* (Farrugia, 2012 ▶); software used to prepare material for publication: *WinGX* (Farrugia, 2012 ▶) and *PLATON* (Spek, 2009 ▶).

## Supplementary Material

Crystal structure: contains datablock(s) I. DOI: 10.1107/S1600536813025324/sj5351sup1.cif

Structure factors: contains datablock(s) I. DOI: 10.1107/S1600536813025324/sj5351Isup2.hkl

Supplementary material file. DOI: 10.1107/S1600536813025324/sj5351Isup3.cml

Additional supplementary materials:  crystallographic information; 3D view; checkCIF report

## Figures and Tables

**Table 1:** Hydrogen-bond geometry (Å, °) *Cg*3 is the centroid of the C2–C7 benzene ring.

*D*—H⋯*A*	*D*—H	H⋯*A*	*D*⋯*A*	*D*—H⋯*A*
O3—H3⋯O4^i^	0.95 (2)	1.64 (2)	2.5793 (15)	170 (2)
C3—H3*A*⋯O3	0.95	2.43	3.367 (2)	168
C9—H9⋯O2^ii^	0.95	2.34	3.275 (2)	170
C14—H14*B*⋯*Cg*3^iii^	0.99	2.85	3.750 (2)	152

Symmetry codes: (i) 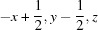; (ii) 
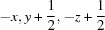
; (iii) 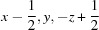.

**Fig. 1.**
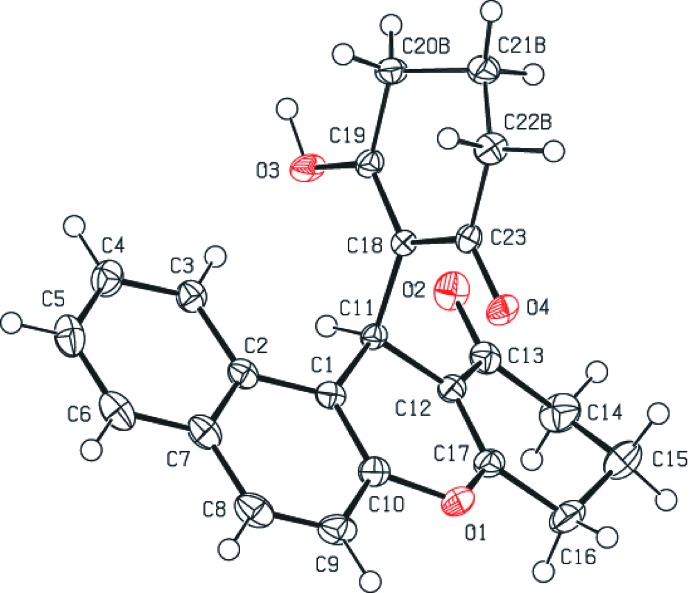
The structure of the title compound with the atom numbering scheme. Displacement ellipsoids for non-H atoms are drawn at the 50% probability level. For clarity only atoms of one disorder component of the disordered methylene groups are shown.

**Fig. 2.**
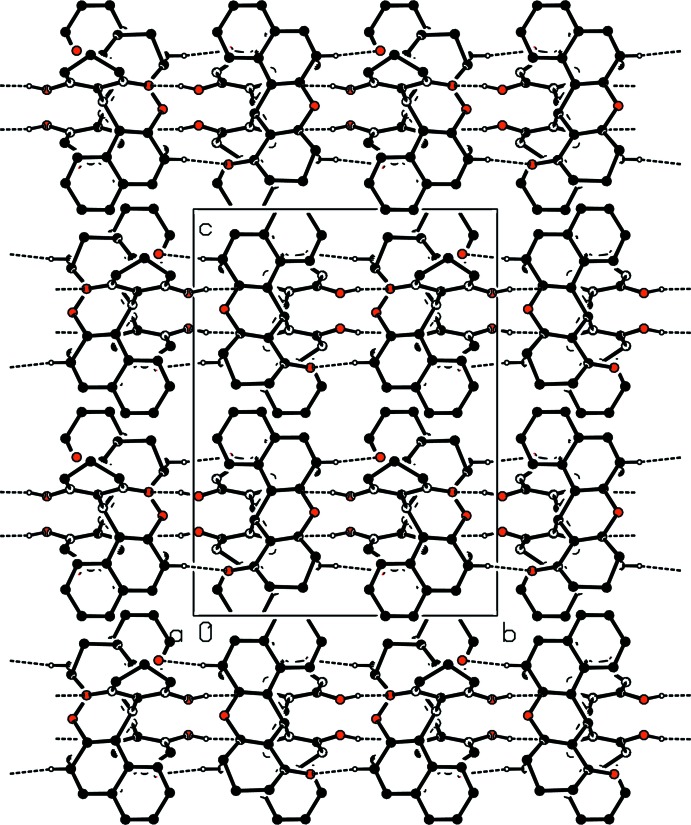
The packing and hydrogen bonding (dashed lines) of the title compound viewing along the a axis. For clarity only atoms of the major disorder component of the disordered methylene groups are shown.

**Fig. 3.**
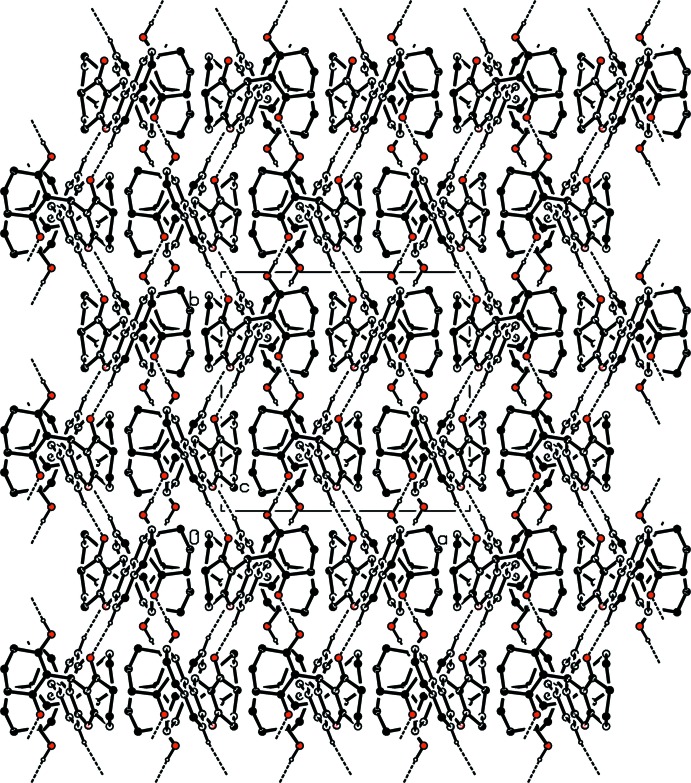
The packing and hydrogen bonding (dashed lines) of the title compound viewing along the c axis. For clarity only atoms of the major disorder component of the disordered methylene groups are shown.

## supplementary crystallographic information

### 1. Comment

Xanthene derivatives have been used as antibacterial, antiviral, antitumor and anti-inflammatory agents (Zare *et al.*, 2012). These compounds also have applications as dyes in laser technology (Menchen *et al.*, 2003*a*,*b*), and as pH sensitive fluorescent materials for the visualization of biomolecules (Sarma & Baruah, 2005). Extending our previous studies of xanthenones (Abdelhamid *et al.*, 2011; Mohamed *et al.*, 2011, 2012), we report herein the synthesis and crystal study of a new of xanthenone derivative.

In the title compound shown in Fig. 1, the pyran ring (O1/C1/C10—C12/C17) has a maximum deviation of 0.111 (1) Å from planarity and the outer cyclohexene ring (C12–C17) of the xanthenone moiety is puckered with the puckering parameters (Cremer & Pople, 1975) of *Q*_T_ = 0.455 (2) Å, θ = 124.4 (2) and φ = 352.8 (3)°. The three methylene C atoms (C20/C21/C22) of the other cyclohexene ring attached to the pyran moiety at atom C11 are disordered over two sets of sites with a ratio of refined occupancies of 0.570 (3): 0.430 (3) and both components of the disordered cyclohexene ring are puckered [puckering parameters: *Q*_T_ = 0.445 (6) Å, θ = 48.7 (6) and φ = 183.8 (10)° for major component (C18/C19/C20B–C22B/C23), and *Q*_T_ = 0.471 (8) Å, θ = 130.2 (8) and φ = 353.3 (13)° for minor component (C18/C19/C20A–C22A/C23)].

The bond lengths in the title compound are within normal ranges (Allen *et al.*, 1987) and are comparable those of similar compounds (Li *et al.*, 2004; Abdelhamid *et al.*, 2011; Çelik *et al.*, 2009; Mohamed *et al.*, 2011, 2012; Reddy *et al.*, 2009).

In the crystal structure, C—H···O and O—H···O hydrogen bonds link the neigbouring molecules (Table 1), forming two dimensional networks parallel to the *ab*-plane (Figs. 2 & 3). A C14—H14B···π interaction also exists between these planes.

### 2. Experimental

The title compound was obtained as the main product during a three component reaction of 1 mmol (206 mg) 4-nitro-2-(trifluoromethyl)aniline, 1 mmol (172 mg) 2-hydroxy-1-naphthaldehyde and 1 mmol (112 mg) 1,3-cyclohexandione in 50 ml ethanol. The reaction mixture was refluxed for 7 h at 351 K. On cooling, the resulting solid was collected, washed with cold ethanol and dried by filtration. The crude product was crystallized by the slow evaporation method over 24 h using ethanol as a solvent. *M*.p. = 517 K, yield = 95%.

### 3. Refinement

The hydroxyl H atoms were found from a difference Fourier map and refined freely. The C-bound H-atoms were refined using a riding model with C—H = 0.95 - 1.00 Å and U_ĩso_(H) = 1.2*U*_eq_(C). The three methylene C atoms (C20/C21/C22) of the other cyclohexene ring bonded to the pyran moiety are disordered over two sets of sites with a ratio of refined occupancies of 0.570 (3): 0.430 (3) [in the refinement, *DFIX* and EADP instructions were used for the disordered atoms].

### Crystal data

**Table:**

C_23_H_20_O_4_	*F*(000) = 1520
*M**_r_* = 360.41	*D*_x_ = 1.325 Mg m^−^^3^
Orthorhombic, *P**b**c**a*	Mo *K*α radiation, λ = 0.7107 Å
Hall symbol: -P 2ac 2ab	Cell parameters from 3838 reflections
*a* = 14.2855 (15) Å	θ = 3.0–29.5°
*b* = 13.7461 (12) Å	µ = 0.09 mm^−^^1^
*c* = 18.400 (2) Å	*T* = 123 K
*V* = 3613.2 (6) Å^3^	Block, colourless
*Z* = 8	0.20 × 0.18 × 0.16 mm

### Data collection

**Table:**

Oxford Diffraction Xcalibur, Eos diffractometer	4541 independent reflections
Radiation source: Enhance (Mo) X-ray Source	3366 reflections with *I* > 2σ(*I*)
Graphite monochromator	*R*_int_ = 0.042
Detector resolution: 16.0727 pixels mm^-1^	θ_max_ = 29.5°, θ_min_ = 3.0°
ω scans	*h* = −18→18
Absorption correction: multi-scan (*CrysAlis PRO*; Oxford Diffraction, 2010)	*k* = −17→17
*T*_min_ = 0.994, *T*_max_ = 1.000	*l* = −16→25
17944 measured reflections	

### Refinement

**Table:**

Refinement on *F*^2^	Primary atom site location: structure-invariant direct methods
Least-squares matrix: full	Secondary atom site location: difference Fourier map
*R*[*F*^2^ > 2σ(*F*^2^)] = 0.051	Hydrogen site location: inferred from neighbouring sites
*wR*(*F*^2^) = 0.114	H atoms treated by a mixture of independent and constrained refinement
*S* = 1.04	*w* = 1/[σ^2^(*F*_o_^2^) + (0.037*P*)^2^ + 1.8151*P*] where *P* = (*F*_o_^2^ + 2*F*_c_^2^)/3
4541 reflections	(Δ/σ)_max_ = 0.001
258 parameters	Δρ_max_ = 0.40 e Å^−^^3^
8 restraints	Δρ_min_ = −0.25 e Å^−^^3^

### Special details

**Table:**

Geometry. Bond distances, angles *etc*. have been calculated using the rounded fractional coordinates. All su's are estimated from the variances of the (full) variance-covariance matrix. The cell e.s.d.'s are taken into account in the estimation of distances, angles and torsion angles

### Fractional atomic coordinates and isotropic or equivalent isotropic displacement parameters (Å^2^)

**Table:**

	*x*	*y*	*z*	*U*_iso_*/*U*_eq_	Occ. (<1)
O1	0.03562 (8)	0.89828 (7)	0.24583 (6)	0.0232 (3)	
O2	0.02801 (9)	0.61517 (8)	0.38998 (6)	0.0269 (4)	
O3	0.18481 (8)	0.51704 (7)	0.29379 (7)	0.0245 (3)	
O4	0.22989 (8)	0.85224 (7)	0.30414 (6)	0.0206 (3)	
C1	0.11053 (11)	0.75665 (10)	0.19287 (8)	0.0170 (4)	
C2	0.14830 (11)	0.71387 (10)	0.12835 (8)	0.0190 (4)	
C3	0.18506 (11)	0.61770 (11)	0.12698 (9)	0.0208 (4)	
C4	0.21973 (12)	0.57841 (12)	0.06413 (9)	0.0259 (5)	
C5	0.21989 (12)	0.63171 (13)	−0.00100 (9)	0.0289 (5)	
C6	0.18492 (12)	0.72413 (12)	−0.00158 (9)	0.0279 (5)	
C7	0.14894 (12)	0.76802 (11)	0.06208 (9)	0.0233 (5)	
C8	0.11215 (13)	0.86400 (11)	0.06150 (9)	0.0275 (5)	
C9	0.07720 (12)	0.90419 (11)	0.12294 (9)	0.0264 (5)	
C10	0.07683 (11)	0.84974 (11)	0.18784 (9)	0.0206 (4)	
C11	0.10633 (10)	0.70142 (9)	0.26465 (8)	0.0150 (4)	
C12	0.04078 (10)	0.75335 (10)	0.31646 (8)	0.0173 (4)	
C13	0.00454 (11)	0.69966 (11)	0.37919 (9)	0.0212 (5)	
C14	−0.06429 (13)	0.75053 (13)	0.42855 (10)	0.0330 (6)	
C15	−0.04989 (15)	0.86009 (13)	0.43098 (11)	0.0370 (6)	
C16	−0.05019 (13)	0.90186 (12)	0.35487 (10)	0.0288 (5)	
C17	0.01241 (11)	0.84547 (11)	0.30557 (9)	0.0205 (4)	
C18	0.20297 (10)	0.68519 (10)	0.29769 (8)	0.0145 (4)	
C19	0.23694 (11)	0.59364 (10)	0.31102 (8)	0.0180 (4)	
C20B	0.3353 (4)	0.5759 (6)	0.3367 (5)	0.0207 (11)	0.570 (3)
C21B	0.3740 (2)	0.66151 (19)	0.37967 (17)	0.0236 (7)	0.570 (3)
C22B	0.3603 (5)	0.7555 (5)	0.3376 (5)	0.0214 (11)	0.570 (3)
C23	0.26030 (11)	0.76828 (10)	0.31416 (8)	0.0160 (4)	
C22A	0.3539 (7)	0.7519 (7)	0.3496 (7)	0.0214 (11)	0.430 (3)
C20A	0.3268 (6)	0.5728 (8)	0.3504 (7)	0.0207 (11)	0.430 (3)
C21A	0.3963 (3)	0.6529 (3)	0.3323 (2)	0.0236 (7)	0.430 (3)
H3A	0.18560	0.58020	0.17030	0.0250*	
H6	0.18470	0.75980	−0.04580	0.0330*	
H4	0.24410	0.51410	0.06460	0.0310*	
H5	0.24410	0.60370	−0.04430	0.0350*	
H3	0.2178 (17)	0.4587 (17)	0.3029 (12)	0.060 (7)*	
H14A	−0.05800	0.72400	0.47830	0.0400*	
H14B	−0.12870	0.73660	0.41160	0.0400*	
H15A	−0.10050	0.89050	0.45990	0.0440*	
H15B	0.01050	0.87490	0.45490	0.0440*	
H16A	−0.11480	0.90080	0.33540	0.0350*	
H16B	−0.02910	0.97040	0.35650	0.0350*	
H20C	0.37600	0.56360	0.29420	0.0250*	0.570 (3)
H20D	0.33630	0.51700	0.36770	0.0250*	0.570 (3)
H21C	0.34160	0.66600	0.42710	0.0280*	0.570 (3)
H21D	0.44160	0.65130	0.38910	0.0280*	0.570 (3)
H22C	0.37900	0.81120	0.36840	0.0260*	0.570 (3)
H22D	0.40120	0.75510	0.29410	0.0260*	0.570 (3)
H8	0.11210	0.90030	0.01760	0.0330*	
H9	0.05310	0.96860	0.12230	0.0320*	
H11	0.07880	0.63600	0.25440	0.0180*	
H20A	0.35200	0.50900	0.33500	0.0250*	0.430 (3)
H20B	0.31560	0.57080	0.40350	0.0250*	0.430 (3)
H21A	0.41270	0.64980	0.28010	0.0280*	0.430 (3)
H21B	0.45420	0.64360	0.36100	0.0280*	0.430 (3)
H22A	0.34670	0.75800	0.40290	0.0260*	0.430 (3)
H22B	0.39770	0.80330	0.33340	0.0260*	0.430 (3)

### Atomic displacement parameters (Å^2^)

**Table:**

	*U*^11^	*U*^22^	*U*^33^	*U*^12^	*U*^13^	*U*^23^
O1	0.0271 (6)	0.0151 (5)	0.0274 (6)	0.0044 (4)	−0.0008 (5)	0.0007 (4)
O2	0.0325 (7)	0.0199 (6)	0.0284 (6)	−0.0053 (5)	0.0035 (5)	0.0024 (5)
O3	0.0248 (6)	0.0096 (5)	0.0392 (7)	0.0003 (4)	−0.0094 (5)	−0.0003 (4)
O4	0.0222 (6)	0.0106 (5)	0.0290 (6)	−0.0015 (4)	0.0001 (5)	0.0000 (4)
C1	0.0156 (7)	0.0143 (7)	0.0212 (8)	−0.0028 (6)	−0.0029 (6)	0.0018 (6)
C2	0.0161 (7)	0.0197 (7)	0.0213 (8)	−0.0045 (6)	−0.0029 (6)	0.0004 (6)
C3	0.0208 (8)	0.0196 (7)	0.0220 (8)	−0.0029 (6)	−0.0009 (7)	−0.0006 (6)
C4	0.0242 (9)	0.0240 (8)	0.0295 (9)	−0.0024 (7)	0.0017 (7)	−0.0052 (7)
C5	0.0264 (9)	0.0385 (10)	0.0219 (8)	−0.0062 (7)	0.0028 (7)	−0.0075 (7)
C6	0.0267 (9)	0.0370 (9)	0.0201 (8)	−0.0090 (7)	−0.0026 (7)	0.0037 (7)
C7	0.0213 (8)	0.0263 (8)	0.0222 (8)	−0.0070 (6)	−0.0033 (7)	0.0027 (7)
C8	0.0309 (10)	0.0265 (8)	0.0251 (9)	−0.0054 (7)	−0.0053 (8)	0.0098 (7)
C9	0.0290 (9)	0.0170 (7)	0.0333 (10)	−0.0004 (6)	−0.0064 (8)	0.0069 (7)
C10	0.0197 (8)	0.0173 (7)	0.0249 (8)	−0.0014 (6)	−0.0026 (7)	0.0000 (6)
C11	0.0158 (7)	0.0105 (6)	0.0186 (7)	−0.0009 (5)	−0.0010 (6)	−0.0002 (5)
C12	0.0129 (7)	0.0171 (7)	0.0220 (8)	−0.0009 (6)	−0.0013 (6)	−0.0028 (6)
C13	0.0168 (8)	0.0231 (8)	0.0236 (8)	−0.0040 (6)	−0.0013 (7)	−0.0018 (6)
C14	0.0263 (10)	0.0400 (10)	0.0326 (10)	0.0039 (8)	0.0110 (8)	0.0010 (8)
C15	0.0332 (11)	0.0389 (10)	0.0390 (11)	0.0124 (8)	0.0068 (9)	−0.0085 (8)
C16	0.0237 (9)	0.0242 (8)	0.0386 (10)	0.0067 (7)	0.0018 (8)	−0.0061 (7)
C17	0.0161 (8)	0.0192 (7)	0.0261 (8)	0.0008 (6)	−0.0027 (7)	−0.0023 (6)
C18	0.0147 (7)	0.0138 (6)	0.0150 (7)	0.0005 (5)	0.0004 (6)	−0.0003 (5)
C19	0.0189 (8)	0.0137 (7)	0.0213 (8)	0.0003 (6)	−0.0010 (6)	−0.0011 (6)
C20B	0.0199 (13)	0.0172 (8)	0.025 (3)	0.0027 (9)	−0.0037 (16)	0.0015 (15)
C21B	0.0171 (12)	0.0240 (11)	0.0297 (13)	0.0017 (9)	−0.0052 (11)	−0.0040 (12)
C22B	0.0152 (11)	0.0190 (9)	0.030 (3)	−0.0042 (9)	0.0005 (15)	−0.0012 (14)
C23	0.0166 (7)	0.0153 (7)	0.0161 (7)	−0.0005 (5)	0.0026 (6)	−0.0006 (6)
C22A	0.0152 (11)	0.0190 (9)	0.030 (3)	−0.0042 (9)	0.0005 (15)	−0.0012 (14)
C20A	0.0199 (13)	0.0172 (8)	0.025 (3)	0.0027 (9)	−0.0037 (16)	0.0015 (15)
C21A	0.0171 (12)	0.0240 (11)	0.0297 (13)	0.0017 (9)	−0.0052 (11)	−0.0040 (12)

### Geometric parameters (Å, º)

**Table:**

O1—C10	1.3894 (19)	C20B—C21B	1.522 (9)
O1—C17	1.3584 (19)	C21A—C22A	1.523 (11)
O2—C13	1.2250 (19)	C21B—C22B	1.519 (8)
O3—C19	1.3281 (18)	C22A—C23	1.505 (11)
O4—C23	1.2469 (17)	C22B—C23	1.503 (8)
O3—H3	0.95 (2)	C3—H3A	0.9500
C1—C10	1.370 (2)	C4—H4	0.9500
C1—C11	1.525 (2)	C5—H5	0.9500
C1—C2	1.431 (2)	C6—H6	0.9500
C2—C3	1.423 (2)	C8—H8	0.9500
C2—C7	1.429 (2)	C9—H9	0.9500
C3—C4	1.369 (2)	C11—H11	1.0000
C4—C5	1.405 (2)	C14—H14A	0.9900
C5—C6	1.365 (2)	C14—H14B	0.9900
C6—C7	1.414 (2)	C15—H15A	0.9900
C7—C8	1.420 (2)	C15—H15B	0.9900
C8—C9	1.354 (2)	C16—H16A	0.9900
C9—C10	1.409 (2)	C16—H16B	0.9900
C11—C18	1.525 (2)	C20A—H20B	0.9900
C11—C12	1.515 (2)	C20A—H20A	0.9900
C12—C13	1.465 (2)	C20B—H20D	0.9900
C12—C17	1.345 (2)	C20B—H20C	0.9900
C13—C14	1.510 (2)	C21A—H21B	0.9900
C14—C15	1.521 (3)	C21A—H21A	0.9900
C15—C16	1.514 (3)	C21B—H21D	0.9900
C16—C17	1.491 (2)	C21B—H21C	0.9900
C18—C23	1.438 (2)	C22A—H22B	0.9900
C18—C19	1.371 (2)	C22A—H22A	0.9900
C19—C20B	1.502 (6)	C22B—H22D	0.9900
C19—C20A	1.502 (10)	C22B—H22C	0.9900
C20A—C21A	1.520 (11)		
			
C10—O1—C17	117.92 (11)	C5—C4—H4	120.00
C19—O3—H3	110.5 (14)	C4—C5—H5	120.00
C2—C1—C11	121.94 (12)	C6—C5—H5	120.00
C10—C1—C11	120.64 (13)	C5—C6—H6	119.00
C2—C1—C10	117.41 (13)	C7—C6—H6	119.00
C1—C2—C7	119.77 (13)	C7—C8—H8	120.00
C3—C2—C7	117.82 (14)	C9—C8—H8	120.00
C1—C2—C3	122.41 (13)	C8—C9—H9	120.00
C2—C3—C4	121.00 (15)	C10—C9—H9	120.00
C3—C4—C5	121.03 (15)	C1—C11—H11	107.00
C4—C5—C6	119.44 (15)	C12—C11—H11	107.00
C5—C6—C7	121.57 (15)	C18—C11—H11	107.00
C2—C7—C6	119.14 (14)	C13—C14—H14A	109.00
C6—C7—C8	121.64 (15)	C13—C14—H14B	109.00
C2—C7—C8	119.21 (14)	C15—C14—H14A	109.00
C7—C8—C9	120.62 (15)	C15—C14—H14B	109.00
C8—C9—C10	119.49 (14)	H14A—C14—H14B	108.00
O1—C10—C9	113.41 (13)	C14—C15—H15A	110.00
C1—C10—C9	123.49 (15)	C14—C15—H15B	110.00
O1—C10—C1	123.06 (14)	C16—C15—H15A	110.00
C1—C11—C18	112.50 (12)	C16—C15—H15B	110.00
C12—C11—C18	112.19 (12)	H15A—C15—H15B	108.00
C1—C11—C12	109.56 (11)	C15—C16—H16A	109.00
C11—C12—C17	122.43 (13)	C15—C16—H16B	109.00
C13—C12—C17	119.04 (14)	C17—C16—H16A	109.00
C11—C12—C13	118.49 (12)	C17—C16—H16B	109.00
O2—C13—C14	121.31 (15)	H16A—C16—H16B	108.00
C12—C13—C14	118.09 (13)	C19—C20A—H20A	110.00
O2—C13—C12	120.58 (14)	C19—C20A—H20B	110.00
C13—C14—C15	112.84 (15)	C21A—C20A—H20A	110.00
C14—C15—C16	110.37 (15)	C21A—C20A—H20B	110.00
C15—C16—C17	111.34 (15)	H20A—C20A—H20B	108.00
O1—C17—C16	111.15 (13)	H20C—C20B—H20D	108.00
C12—C17—C16	125.43 (15)	C19—C20B—H20D	109.00
O1—C17—C12	123.39 (14)	C21B—C20B—H20C	109.00
C11—C18—C19	121.74 (13)	C19—C20B—H20C	109.00
C19—C18—C23	119.33 (13)	C21B—C20B—H20D	109.00
C11—C18—C23	118.92 (12)	C20A—C21A—H21B	110.00
O3—C19—C18	119.12 (14)	C22A—C21A—H21A	110.00
O3—C19—C20A	116.3 (4)	C20A—C21A—H21A	110.00
C18—C19—C20B	122.4 (3)	H21A—C21A—H21B	108.00
C18—C19—C20A	124.3 (4)	C22A—C21A—H21B	110.00
O3—C19—C20B	118.1 (3)	C20B—C21B—H21D	110.00
C19—C20A—C21A	108.3 (7)	C20B—C21B—H21C	110.00
C19—C20B—C21B	112.2 (5)	H21C—C21B—H21D	108.00
C20A—C21A—C22A	110.0 (6)	C22B—C21B—H21C	110.00
C20B—C21B—C22B	110.3 (5)	C22B—C21B—H21D	110.00
C21A—C22A—C23	113.4 (7)	C23—C22A—H22B	109.00
C21B—C22B—C23	111.6 (5)	H22A—C22A—H22B	108.00
C18—C23—C22A	118.6 (4)	C21A—C22A—H22A	109.00
O4—C23—C22A	120.8 (4)	C21A—C22A—H22B	109.00
C18—C23—C22B	120.6 (3)	C23—C22A—H22A	109.00
O4—C23—C18	120.38 (14)	C21B—C22B—H22C	109.00
O4—C23—C22B	118.8 (3)	C21B—C22B—H22D	109.00
C2—C3—H3A	120.00	C23—C22B—H22C	109.00
C4—C3—H3A	119.00	C23—C22B—H22D	109.00
C3—C4—H4	119.00	H22C—C22B—H22D	108.00
			
C17—O1—C10—C1	−12.4 (2)	C18—C11—C12—C13	−72.26 (16)
C17—O1—C10—C9	165.31 (14)	C18—C11—C12—C17	110.02 (16)
C10—O1—C17—C12	11.4 (2)	C1—C11—C18—C19	−120.34 (15)
C10—O1—C17—C16	−166.72 (13)	C1—C11—C18—C23	58.68 (17)
C10—C1—C2—C3	−179.85 (14)	C12—C11—C18—C19	115.59 (15)
C10—C1—C2—C7	−0.5 (2)	C12—C11—C18—C23	−65.40 (17)
C11—C1—C2—C3	−0.6 (2)	C11—C12—C13—O2	1.1 (2)
C11—C1—C2—C7	178.76 (14)	C11—C12—C13—C14	−177.18 (14)
C2—C1—C10—O1	177.82 (14)	C17—C12—C13—O2	178.93 (15)
C2—C1—C10—C9	0.4 (2)	C17—C12—C13—C14	0.6 (2)
C11—C1—C10—O1	−1.5 (2)	C11—C12—C17—O1	3.6 (2)
C11—C1—C10—C9	−178.93 (15)	C11—C12—C17—C16	−178.61 (15)
C2—C1—C11—C12	−164.88 (14)	C13—C12—C17—O1	−174.15 (14)
C2—C1—C11—C18	69.61 (17)	C13—C12—C17—C16	3.7 (2)
C10—C1—C11—C12	14.38 (19)	O2—C13—C14—C15	152.03 (16)
C10—C1—C11—C18	−111.14 (16)	C12—C13—C14—C15	−29.7 (2)
C1—C2—C3—C4	179.23 (15)	C13—C14—C15—C16	53.5 (2)
C7—C2—C3—C4	−0.1 (2)	C14—C15—C16—C17	−48.5 (2)
C1—C2—C7—C6	−178.84 (15)	C15—C16—C17—O1	−160.62 (14)
C1—C2—C7—C8	0.2 (2)	C15—C16—C17—C12	21.3 (2)
C3—C2—C7—C6	0.5 (2)	C11—C18—C19—O3	0.9 (2)
C3—C2—C7—C8	179.57 (15)	C11—C18—C19—C20B	173.9 (4)
C2—C3—C4—C5	−0.2 (2)	C23—C18—C19—O3	−178.09 (14)
C3—C4—C5—C6	0.0 (3)	C23—C18—C19—C20B	−5.1 (5)
C4—C5—C6—C7	0.4 (3)	C11—C18—C23—O4	2.7 (2)
C5—C6—C7—C2	−0.7 (3)	C11—C18—C23—C22B	−172.0 (4)
C5—C6—C7—C8	−179.71 (17)	C19—C18—C23—O4	−178.22 (14)
C2—C7—C8—C9	0.3 (3)	C19—C18—C23—C22B	7.0 (4)
C6—C7—C8—C9	179.32 (17)	O3—C19—C20B—C21B	−159.1 (4)
C7—C8—C9—C10	−0.5 (3)	C18—C19—C20B—C21B	27.9 (7)
C8—C9—C10—O1	−177.54 (15)	C19—C20B—C21B—C22B	−50.8 (7)
C8—C9—C10—C1	0.1 (3)	C20B—C21B—C22B—C23	52.4 (6)
C1—C11—C12—C13	162.05 (13)	C21B—C22B—C23—O4	153.7 (4)
C1—C11—C12—C17	−15.67 (19)	C21B—C22B—C23—C18	−31.5 (7)

### Hydrogen-bond geometry (Å, º)

Cg3 is the centroid of the C2–C7 benzene ring.

**Table:**

*D*—H···*A*	*D*—H	H···*A*	*D*···*A*	*D*—H···*A*
O3—H3···O4^i^	0.95 (2)	1.64 (2)	2.5793 (15)	170 (2)
C3—H3*A*···O3	0.95	2.43	3.367 (2)	168
C9—H9···O2^ii^	0.95	2.34	3.275 (2)	170
C11—H11···O3	1.00	2.34	2.8228 (17)	108
C14—H14*B*···*Cg*3^iii^	0.99	2.85	3.750 (2)	152

Symmetry codes: (i) −*x*+1/2, *y*−1/2, *z*; (ii) −*x*, *y*+1/2, −*z*+1/2; (iii) *x*−1/2, *y*, −*z*+1/2.
